# Green synthesis of carbon-supported nanoparticle catalysts by physical vapor deposition on soluble powder substrates

**DOI:** 10.1038/srep14245

**Published:** 2015-09-18

**Authors:** Hee-Young Park, Injoon Jang, Namgee Jung, Young-Hoon Chung, Jae Yoon Ryu, In Young Cha, Hyung Juhn Kim, Jong Hyung Jang, Sung Jong Yoo

**Affiliations:** 1 Fuel Cell Research Center, Korea Institute of Science and Technology (KIST), Seoul 136-791, Republic of Korea; 2 Graduate School of Energy Science and Technology (GEST), Chungnam National University, Daejeon 305-764, Republic of Korea; 3 School of Chemical and Biological Engineering, Seoul National University (SNU), Seoul 151-742, Republic of Korea

## Abstract

Metal and metal oxide nanoparticles (NPs) supported on high surface area carbon (NP/Cs) were prepared by the physical vapor deposition of bulk materials on an α-D-glucose (Glu) substrate, followed by the deposition of the NPs on carbon supports. Using Glu as a carrier for the transport of NPs from the bulk materials to the carbon support surfaces, ultrafine NPs were obtained, exhibiting a stabilizing effect through OH moieties on the Glu surfaces. This stabilizing effect was strong enough to stabilize the NPs, but weak enough to not significantly block the metal surfaces. As only the target materials and Glu are required in our procedure, it can be considered environmentally friendly, with the NPs being devoid of hazardous chemicals. Furthermore, the resulting NP/Cs exhibited an improvement in activity for various electrochemical reactions, mainly attributed to their high surface area.

In the past three decades, fine metal or metal oxide nanoparticles (NPs) have been extensively investigated because of their unique properties attributed to a high surface/bulk ratio and a finite number of atoms in the particles. However, the thermodynamic instability of NPs, which originates from their finite size, hinders their use in practical applications. Hence, NPs supported on high surface area materials, which enhance their stability, are widely used for industrial applications, such as catalysis, and in energy generation or energy storage devices and sensors. In particular, NPs supported on high surface area carbon (NP/C) have attracted increasing interest for application in electrical energy generation and storage systems. For example, hydrogen-air fuel cells, which are promising electrical power sources that do not emit CO_2_, utilize carbon-supported Pt NPs (Pt/C) for catalyzing the fuel cell reactions^1^.

Carbon-supported NPs can be prepared by the chemical and/or physical reduction of metal ions. Typically, impregnation^2^ is widely used for preparing supported NPs. Meanwhile, with the use of microemulsion or microwave irradiation, NPs can be obtained with a narrow and controllable size distribution^2^. However, these approaches require expensive reagents, such as metal ion complexes, solvents, and stabilizing or reducing agents, which often exhibit potential environmental and biological hazards^3^.

Meanwhile, fine metal NPs have been prepared by the physical vapor deposition (PVD) of corresponding metals, in the absence of metal complexes. PVD on liquid substrates, such as ionic liquids (ILs)^4^,^5^,^6^,^7^,^8^,^9^, vegetable oils^6^, and liquid N_2_-cooled acetone^10^ has been reported for the preparation of NPs with a tunable composition and size distribution. Examples include the Au–Ag alloy (Au/Ag ratio of 0–1) by Okazaki *et al.*^8^ and the Au–Cu alloy (Au/Cu ratio of 0–1) by König *et al.*^5^ Recently, Hernandez-Fernandez *et al.* reported the use of PVD to prepare Pt_x_Y alloy NPs on a glassy carbon substrate using a bulk Pt_9_Y alloy^11^, as the chemical approach did not yield Pt_x_Y alloy NPs, due to the high affinity of Y for oxygen. PVD is therefore believed to be an alternative to chemical approaches, which can potentially be extended to the synthesis of NP catalyst materials.

The use of NPs in practical applications requires their deposition on high surface area supports. However, preparation of supported NPs by PVD remains a challenge. In addition, a few studies have reported the synthesis of Pt NPs on high surface area Al_2_O_3_^12^,^13^. However, for electrochemical systems, the NPs should be deposited on conductive materials, such as high surface area carbon materials, carbon nanotubes (CNT), and semiconducting materials (TiO_2_)^14^,^15^,^16^.

We herein describe a general, clean, and economical strategy for the preparation of NP/Cs. This strategy consists of: 1) The deposition of NPs on α-D-glucose powder (NP/Glu) by PVD; and 2) Transfer of the NPs from Glu to carbon supports using a mixture of NP/Glu, high surface area carbon support, and solvent. This strategy will also be extended to prepare NPs on a range of supports, such as carbon nanotubes (CNTs), graphene oxide (GO), and TiO_2_, thereby demonstrating its generality. PVD will be employed for the facile preparation of various types of NPs on the surface of Glu powder, including noble metals, alloys, and transition-metal oxides. The prepared NPs are expected to exhibit a finite size and narrow size distribution. Moreover, our NPs will not contain surfactants, which often impede catalytic reactions, as the NPs will be directly prepared using the corresponding bulk materials. Due to Glu’s abundance and non-toxic nature, it was selected as the soluble powder substrate for transferring our NPs from the bulk target to the high surface area carbon supports. Furthermore, it can be readily removed from NP surfaces whilst also stabilizing the NPs. The use of Glu as a carrier solves several issues inherent with conventional NP synthesis^2^,^3^,^4^, such as the use of harmful and/or expensive reagents (chemical approaches), difficulties in removing residual ILs from NP surfaces (PVD on ILs), and the additional equipment required for PVD on liquid nitrogen-cooled acetone. PVD on Glu powder therefore appears a promising approach for NP/C preparation.

## Results and Discussion

**Figure 1:**
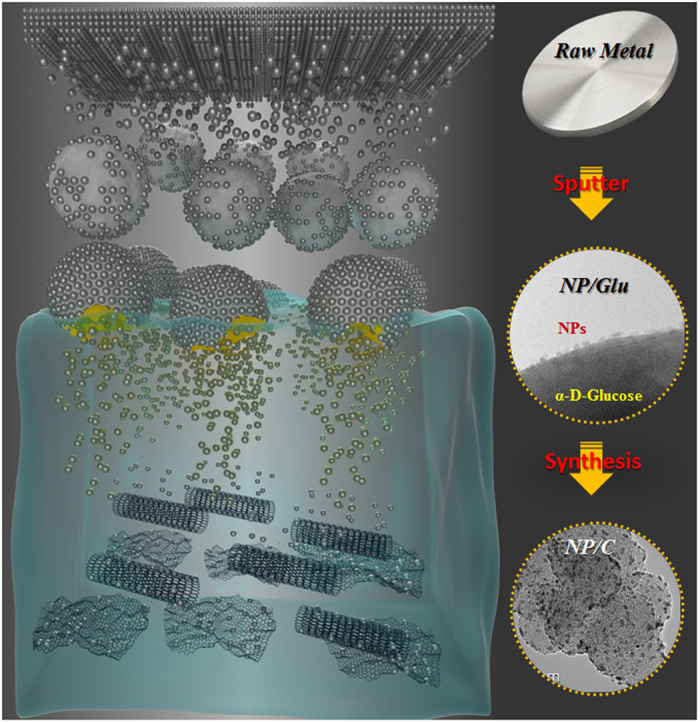
Schematic representation of NP/C synthesis.

Figure 1 shows a schematic diagram representing the synthesis of NP/Cs. The NPs were first deposited on Glu by the PVD of the corresponding bulk materials (Supplementary Fig. 1 online) using specially designed apparatus (Supplementary Fig. 2 online). The resulting NP/Glu was mixed with a carbon dispersion in either water or ethanol, and the mixture filtered, washed, and dried in an oven to give the desired NP/Cs.

**Figure 2:**
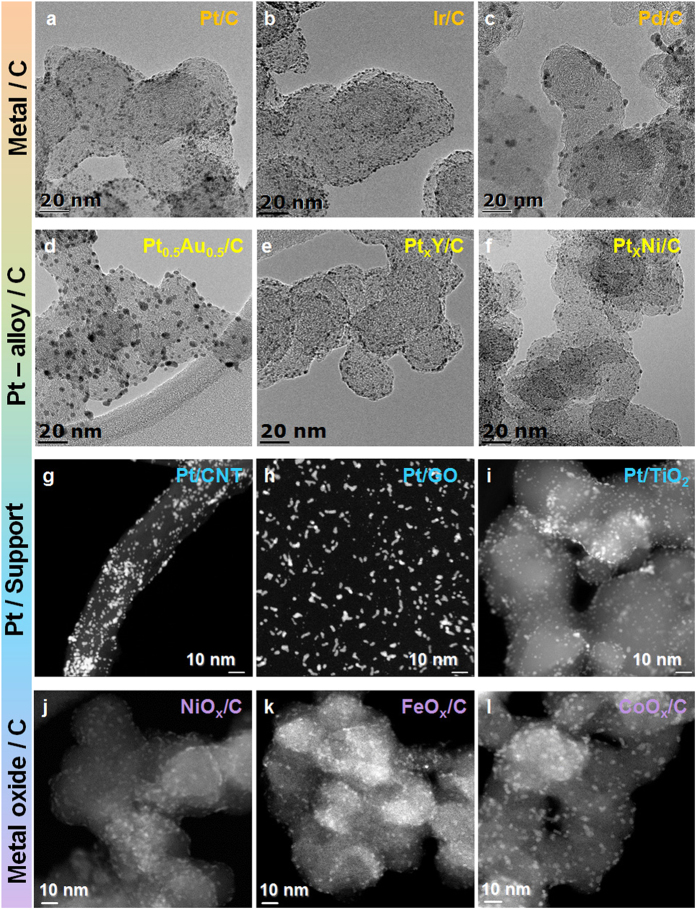
TEM images of NPs deposited on various high surface area supports prepared using NP/Glu. (**a**) Pt/C, (**b**) Ir/C, (**c**) Pd/C, (**d**) Pt_0.5_Au_0.5_/C, (**e**) Pt_x_Y/C, (**f**) Pt_x_Ni/C, (**g**) Pt/CNT, (**h**) Pt/GO, (**i**) Pt/TiO_2_, (**j**) NiO_x_/C, (**k**) FeO_x_/C, and (**l**) CoO_x_/C.

Figure 2 shows typical transmission electron microscopy (TEM) images of the prepared NP/supports. Carbon-supported Pt nanoparticles (Pt/C), prepared using Pt/Glu, showed finite-sized (~2 nm) Pt NPs with a narrow size distribution, which is essential for high activity (Fig. 2a). The size and shape of Pt NPs prepared at higher scale (around 500 g of glucose) were fairly maintained (Supplementary Fig. 3 online). The Pt NP size was affected by the sputtering conditions, such as working pressure, sputtering power, and distance between the Pt target and Glu substrate (Supplementary Fig. 4 online). Using the same method, well-dispersed Ir/C (~1.5 nm) and Pd/C (~4 nm) NP/Cs were also prepared (Fig. 2b,c, and Supplementary Fig. 5 online).

This strategy was also extended to the formation of Pt alloy NPs, prepared by NP co-deposition, as König *et al.* suggested that co-deposition is effective for the preparation of alloy NPs^5^. The TEM images of Pt_0.5_Au_0.5_/C (Fig. 2d) show that small alloy NPs (~5 nm) were prepared by the co-deposition of Pt and Au, with the size of the Pt_x_Au_1_−_x_ NPs increasing with higher Au content (Supplementary Fig. 5 online). From the X−ray diffraction (XRD) pattern of Pt_x_Au_1−x_/C (Supplementary Fig. 6 online) and energy-dispersive X-ray spectroscopic (EDX) analysis of Pt_0.5_Au_0.5_/C (Supplementary Fig. 7 online), it was confirmed that a well-mixed alloy was prepared, with the atomic composition of the alloy easily adjusted by control of the Pt and Au deposition rates. The XRD patterns of Pt_x_Y/C and Pt_x_Ni/C (highly efficient catalysts for fuel cell reactions^11^) indicated the formation of Pt alloys (Supplementary Figs. 8 online), while TEM images of Pt_x_Y/C or Pt_x_Ni/C (Fig. 2e,f), also showed an even distribution of particles on the carbon support surfaces without agglomeration.

This approach was not limited to the high surface area carbon support; various supports such as CNTs, graphene oxide (GO), and TiO_2_ were utilized, as shown in the TEM images of Pt on various supports (Fig. 2g−i). Considering that deposition of Pt NPs on CNTs often requires anchor sites on the CNT surfaces, such as defects or molecular linkers, Pt/Glu was suggested for the preparation of Pt/CNTs. This route avoided damage to the CNTs, as the formation of defects, and the use of complex chemical reactions for forming molecular linkages were not required. Furthermore, Pt/GO and Pt/TiO_2_ were also prepared using the Pt/C procedure. Pt NP size was largely unaffected by the different supports, indicating that the NPs in Pt/Glu were transferred to the support surfaces without loss or aggregation of the NPs, although further investigation for the deposition mechanism is needed.

Our approach also provided carbon-supported fine metal oxide NPs (MO/C). Carbon-supported Co oxide NPs (CoO_x_/C) exhibited a fine size distribution and dispersion (Fig. 2j). The CoO_x_ NPs measured approximately 3 nm, which is, to the best of our knowledge, the smallest reported carbon-supported CoO_x_ nanoparticle. Notably, our approach also allowed the preparation of ultrafine metal oxide NPs in the absence of strong organic surfactants. PVD on Glu was used to produce NiO_x_/C and FeO_x_/C exhibiting ultrafine size, and evenly dispersed NPs on carbon supports (Fig. 2k,l, and Supplementary Fig. 9 online).

**Figure 3:**
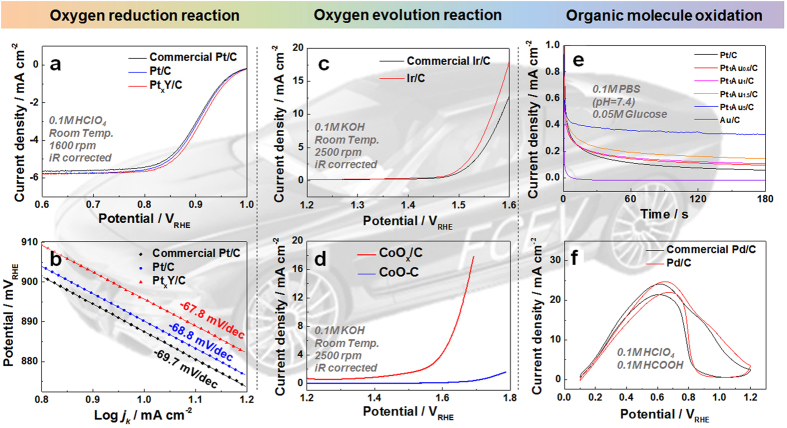
Evaluation of NP/C electrochemial activity. (**a**) Polarization curves and (**b**) Tafel plots for ORR on Pt/C, Pt_x_Y/C, and commercial Pt/C. (**c**) OER polarization curves of Ir/C and commercial Ir/C. (**d**) OER polarization curves of Co_x_O/C and commercial CoO-C. (**e**) Chronoamperometry curve for the glucose oxidation reaction on Pt_x_Au_1−x_/C (0 ≤ x ≤ 1). (**f**) Polarization curves for FOR on Pd/C and commercial Pd/C.

NP/Cs are expected to exhibit high activity, primarily attributed to their high active surface area (see Supplementary Table1). To confirm this, the electrochemical activity of our NP/Cs was examined for a number of reactions (Fig. 3a–f). Pt/C exhibited oxygen reduction reaction (ORR) activity comparable to the commercial Pt/C catalyst (Fig. 3a), while Pt_x_Y/C exhibited an improvement in ORR activity (Supplementary Fig. 11 online) due to its high surface area and alloy with Y. A similar phenomenon was reported by Hernandez−Fernandez *et al.* using size-selected Pt_x_Y NPs on a glassy carbon substrate. Figure 3b shows the Tafel slopes of the ORRs of Pt/C (69.7 mV dec^−1^) and PtY/C (67.8 mV dec^−1^), suggesting that the synthesized catalysts and commercial Pt/C catalysts have the same reaction mechanisms. Ir/C also showed improved oxygen evolution reaction (OER) activity (1.45− fold at 1.55 V) compared to commercial Ir/C (Fig. 3c), mainly due to the increased electrochemical surface area (1.15−fold), estimated using the hydrogen desorption charge from the CV curve (Supplementary Fig. 12 online). Our developed method yields a number of advantages, observed upon the examination of MO/C catalysts. CoO_x_/C exhibited a 42.6-fold enhancement (at 1.65 V) in the OER activity (Fig. 3d) compared with commercial CoO deposited on a thin film of carbon support (CoO-C), likely due to the high electrochemically active surface area (EASA) of CoO_x_ in CoO_x_/C (231 m^2^ g^−1^, see Supplementary Table 2 online). The specific activities of Pt/C, Ir/C, and CoO_x_/C were comparable with the corresponding commercial catalysts (Supplementary Fig. 15 online), suggesting that reaction kinetics were largely unaffected by any residual glucose. Variation in the atomic compositions of Pt and Au resulted in significant changes in the PtAu/C glucose oxidation reaction activity (Fig. 3e) in accordance with previous studies^17^,^18^, indicating that PtAu NPs can be prepared in a wide range of atomic compositions. In addition, in the formic acid oxidation reaction (FOR), Pd/C and commercial Pd/C showed comparable activity (Fig. 3f), while the EASA of Pd/C was lower than that of commercial Pd/C. It was therefore speculated that residual glucose on the Pd surfaces was advantageous for the FOR kinetics.

**Figure 4:**
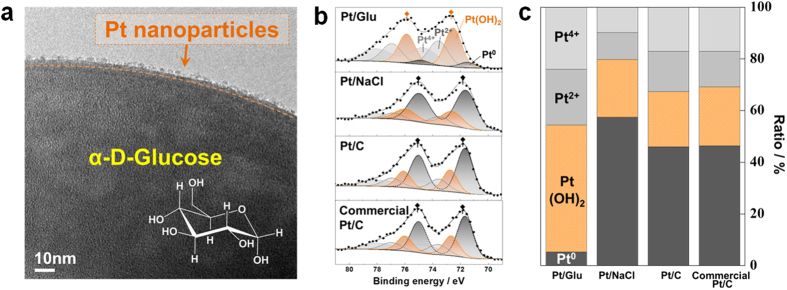
Identification of Glu stabilization effect on Pt NPs. (**a**) TEM image of Pt/Glu. (**b**) Pt 4f XPS diagrams of Pt/Glu, Pt/NaCl, Pt/C, and commercial Pt/C. (**c**) Area ratio derived from the curve fitting of Pt 4f XPS of Pt/Glu, Pt/NaCl, Pt/C, and commercial Pt/C.

To identify the stabilizing effect of Glu on the size and structure of NPs deposited by PVD, Pt/Glu and Pt NPs on NaCl powder (Pt/NaCl) were prepared using equally sized Glu and NaCl powders (NaCl was selected as an example of a soluble powder substrate having little interaction with the Pt NPs). As shown in the cryo-TEM images of Pt/Glu in Fig. 4a, separated Pt nanoparticles of approx. 2 nm were observed (Fig. 4a), corresponding to the size of Pt NPs in Pt/C, while significant growth of Pt NP_S_ (>10 nm) was observed in Pt/NaCl (Supplementary Fig. 13 online). From the X-ray photoelectron spectroscopy (XPS) curves of Pt/Glu and Pt/NaCl, the Pt−OH moiety was more abundant in Pt/Glu, indicating significant interactions between Pt and Glu. In contrast, comparable Pt 4f XPS curves were obtained for Pt/NaCl and commercial Pt/C, suggesting little interactions between Pt NPs and NaCl (Fig. 4b,c, and Supplementary Fig. 14 online). As Pt/Glu and Pt/NaCl were prepared according to the same method, the −OH moieties were considered to originate from Glu. The surface concentrations of Pt in Pt/Glu and Pt/NaCl were virtually identical, and thus the Glu substrate was expected to stabilize the Pt NPs. However, this was not observed for the NaCl substrate, which exhibited little stabilization. The stabilizing effect of the OH moieties was also reported by Wender *et al.* in sputtered Ag/vegetable oil systems^6^.

Pt−OH was easily removed from the Pt surfaces by washing with water, resulting in little significant difference between the XPS of commercial Pt/C (Premertek) and the Pt/C prepared herein. Raveendran *et al.* reported that the binding interactions between Pt and OH are weaker than those between Pt and thiols or Pt and amines^3^, and so the OH group in Glu can stabilize the Pt NPs without the toxic effects of thiols or amines^3^. In addition, a significant amount of Glu molecules were observed on the MO/C surfaces, confirmed by a corresponding weight loss at 200–300 °C in thermogravimetric analysis (data not shown), suggesting glucose combustion. The presence of Glu on MO/C is likely attributed to the strong interactions between the OH moiety and the MO surfaces. However, the Glu moieties on MO were readily removed by calcination at 180 °C for 1 h in air, confirmed by no observed weight loss at ~200–300 °C (Supplementary Fig. 18 online). Thus, the MO/Cs utilized in this study were subjected to calcination at 180 °C for 1 h.

In summary, we have described a novel strategy for preparing NP/Cs with a finite particle size and corresponding high surface area. Various NP/Cs, based on noble metals, alloys, and transition-metal oxides, were prepared. The reported NP/Cs exhibited improved activities, mainly attributed to their high surface areas. This strategy was further extended to the preparation of various NP/support systems, including Pt/CNT, Pt/GO, and Pt/TiO_2_. The use of glucose as a carrier for transferring NP from bulk metals to the carbon support surfaces led to the formation of ultrafine nanoparticles with a stabilizing effect, due to the presence of OH moieties on the Glu surfaces. This stabilizing effect was strong enough to stabilize Pt NPs, and weak enough to ensure that Pt/C surfaces were not blocked to incoming reagents for the above reactions.

## Methods

### Preparation of NP/Glu

Glu powder (Sigma-Aldich, anhydrous, 96%) was added to the reaction vessel, and the Glu powder stirred during the NPs deposition (Supplementary Fig. 1). The metal (Pt, Ir, Pd) or metal oxide (NiO, FeO, CoO) NPs were directly deposited on the Glu powder using a radio-frequency (RF) magnetron sputter system with a 4 in metal or metal oxide target (United Vacuum & Materials, 99.99%) under an Ar atmosphere. The Pt-alloy NPs were deposited by a co-sputter technique using Pt and alloy metals (Au, Ni, and Y, United Vacuum & Materials, 99.99%). The base pressure was <2 × 10^−6^ Torr, and the working pressure was maintained at 5 mTorr during sputtering. The sputtering powers for the NP/Glu preparation were given in the Supplementary Table 3. The working distance between the sputter target and the substrate was 20 cm.

### Synthesis of NP/C

For the preparation of the M/Cs, high surface area carbon (Vulcan XC−72 R, Cabot Co., BET: 237 m^2^/g, denoted as C) was added to purified DI water (250 mL) in a flask and sonicated at room temperature for 30 min. A calculated amount of M/Glu was then dissolved in the above solution, and the resulting solution stirred for 3 h at 80 °C, and aged for ~12 h at room temperature. Subsequently, the product was thoroughly washed with DI water and dried overnight at 60 °C. Pt/GO and Pt/TiO_2_ were also prepared according to this procedure, replacing C with GO (Carbon convergence material research center, KIST Jeonbuk branch composite material technology institute), and TiO_2_ nanopowder (Sigma Aldrich, particle size <100 nm). To prepare MO/C and Pt/CNT, high surface area carbon and multi-wall CNTs (CNT MR99, Carbon Nano-material Technology Co., LTD, Republic of Korea), were dispersed in EtOH (250 mL). The CNTs were used without further purification. A calculated amount of MO/Glu (or Pt/Glu for Pt/CNT) was then added, and the resulting solution stirred overnight (~12 h). The solution was filtered and washed with DI water, and the resulting product was dried at 60 °C to obtain MO/C and Pt/CNT. Finally, the MO/C was calcined at 180 °C for 1 h in air.

### Physical characterization

TEM images were recorded on an FEI Titan^TM^ 80–300 microscope operated at 300 kV. EDX elemental analysis was carried out using a JEOL ARM200F microscope. The cryo-TEM measurements were conducted on an FEI Tecnai F20 G^2^ microscope operated at 200 kV. XPS analysis was performed on a Multilab ESCA 2000 from VG Microtech using Al Kα as the X-ray radiation source. Before measurements were carried out, the binding energy scale was calibrated using corresponding photoelectron lines of Ag 3d 5/2 at 368.21 eV. XRD measurements were conducted on a Dmax2500/PC (Rigaku Co.) with Cu Kα as the X-ray radiation source (k = 1.54056 Å) operating at 40 kV and 200 mA, scanning 2θ between 20° and 90° at 2° min^−1^.

### Electrochemical analysis

Electrochemical experiments were conducted using an Autolab PGSTAT 302 N potentiostat, controlled by a computer. A standard three-compartment glass cell was used for all experiments, and the measurements were conducted at room temperature (see Supplementary Information for detailed procedures). The working electrode was a thin layer of a Nafion-impregnated catalyst cast on a glassy carbon disc, held in a Teflon cylinder. Pt wire and the saturated calomel electrode (SCE) were used as the counter and reference electrodes, respectively. All potentials in the manuscript are quoted with respect to the reversible hydrogen electrode (RHE).

## Additional Information

**How to cite this article**: Park, H.-Y. *et al.* Green synthesis of carbon-supported nanoparticle catalysts by physical vapor deposition on soluble powder substrates. *Sci. Rep.* **5**, 14245; doi: 10.1038/srep14245 (2015).

## Supplementary Material

Supplementary Information
