# Corrigendum: Green synthesis of carbon-supported nanoparticle catalysts by physical vapor deposition on soluble powder substrates

**DOI:** 10.1038/srep20745

**Published:** 2016-02-08

**Authors:** Hee-Young Park, Injoon Jang, Namgee Jung, Young-Hoon Chung, Jaeyune Ryu, In Young Cha, Hyoung-Juhn Kim, Jong Hyun Jang, Sung Jong Yoo

Scientific Reports 5: Article number: 1424510.1038/srep14245; published online: 09182015; updated: 02082016.

The original version of this Article contained typographical errors in the spelling of the authors Jaeyune Ryu, Hyoung-Juhn Kim and Jong Hyun Jang, which were incorrectly given as Jae Yoon Ryu, Hyung Juhn Kim and Jong Hyung Jang. These errors have now been corrected in the PDF and HTML versions of the Article.

