# Supplementary material for: Green synthesis of carbon-supported nanoparticle catalysts by physical vapor deposition on soluble powder substrates
Source: Sci Rep. 2015 Sep 18;5:14245. doi: 10.1038/srep14245 (PMC4585564; doi:10.1038/srep14245)
Supplement: Supplementary Information [file srep14245-s1.pdf]

Supplementary Information

Green synthesis of carbon-supported nanoparticle catalysts by physical vapor deposition on soluble powder substrate

Hee-Young Park[1,†], Injoon Jang[1, †], Namgee Jung[2], Young-Hoon Chung[1], Jae Yoon Ryu[1], In-Young Cha[1], Hyung Juhn Kim[1], Jong Hyung Jang[1], Sung Jong Yoo[1,*]

Fuel Cell Research Center, Korea Institute of Science and Technology (KIST), Seoul 136-791, Republic of Korea.

Graduate School of Energy Science and Technology (GEST), Chungnam National University, Daejeon 305-764, Republic of Korea.

Correspondence should be addressed to S. J. Yoo,

Fax: +82-958-5199, Tel.: +82-2-958-5260, E-mail: ysj@kist.re.kr

[*]These authors contributed equally to this work.

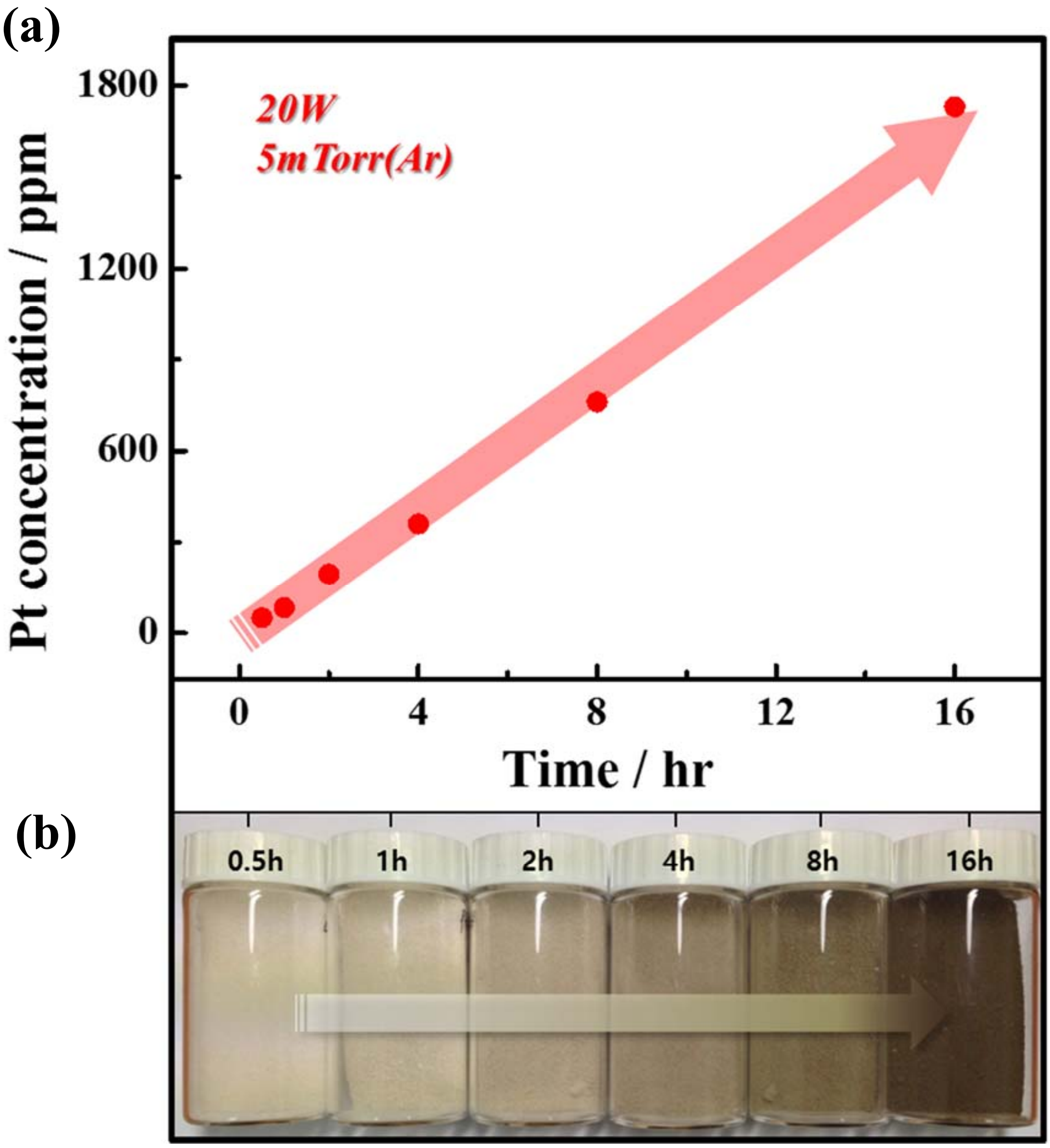

Supplementary fig. 1. Pt concentration of prepared Pt/Glu. (a) ICP analysis of Pt/Glucose indicated that the Pt concentration in Pt/Glu linearly increased with the increased deposition time (b) Color variation of Pt/Glu in accordance with increasing Pt concentration (deposition time).

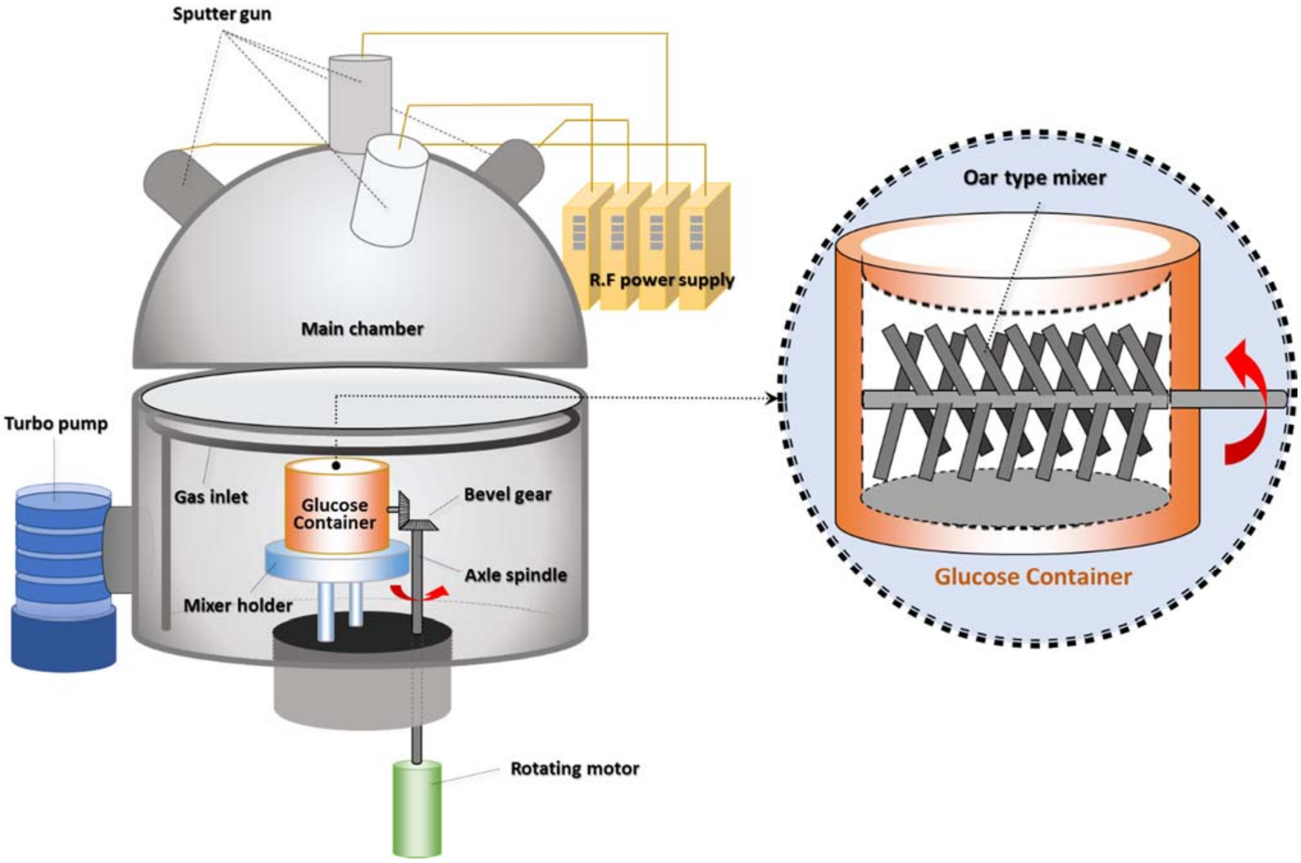

Supplementary Fig. 2. Schematic representation of the sputter system with specially designed container for mixing the glucose powder during PVD process.

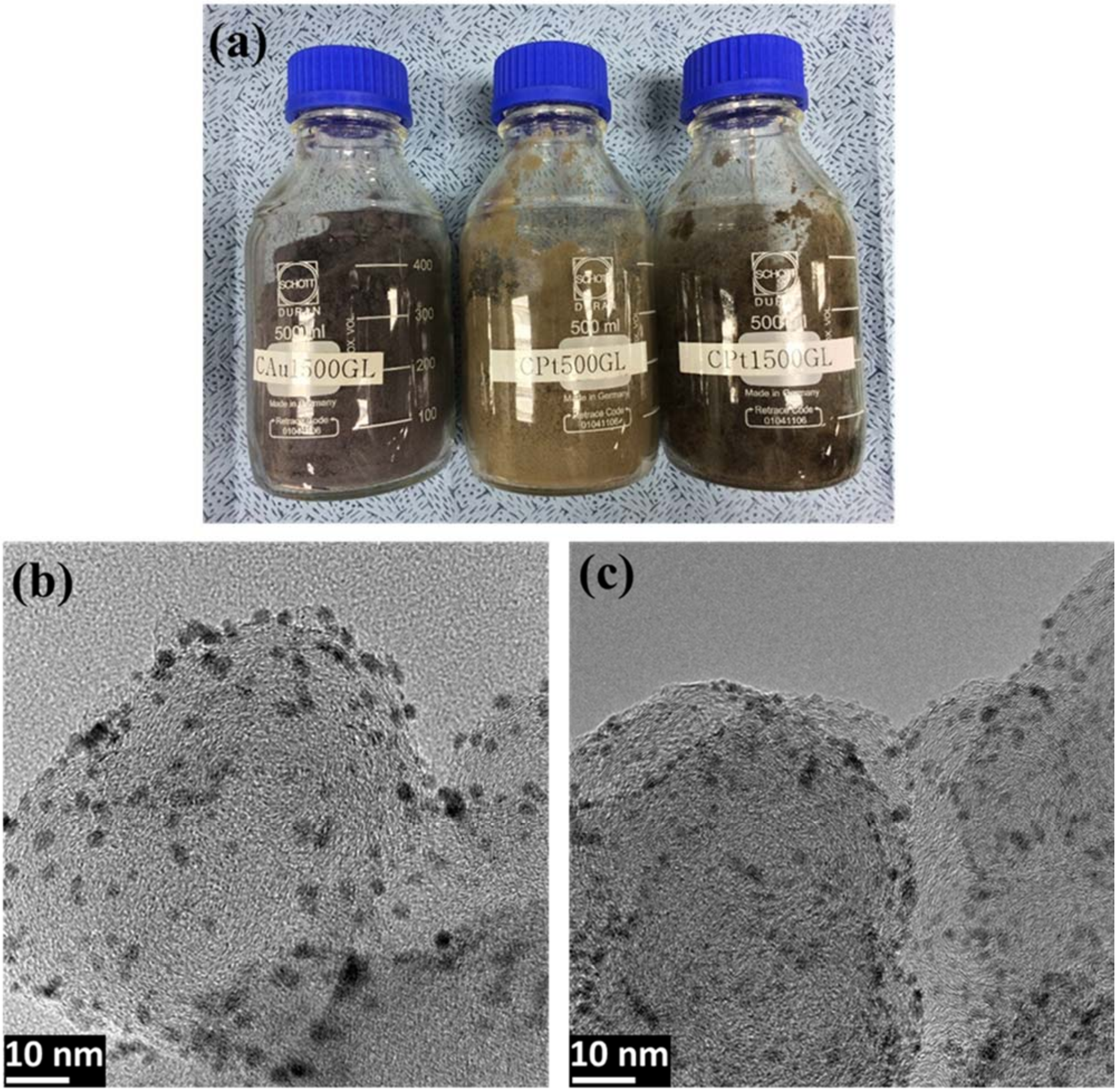

Supplementary fig. 3. (a) Photo of Au/Glu and Pt/Glu. Transmission electron microcopy (TEM) images of Pt/C prepared using Pt/Glu of (b) 500 g scale and (c) 40 g scale.

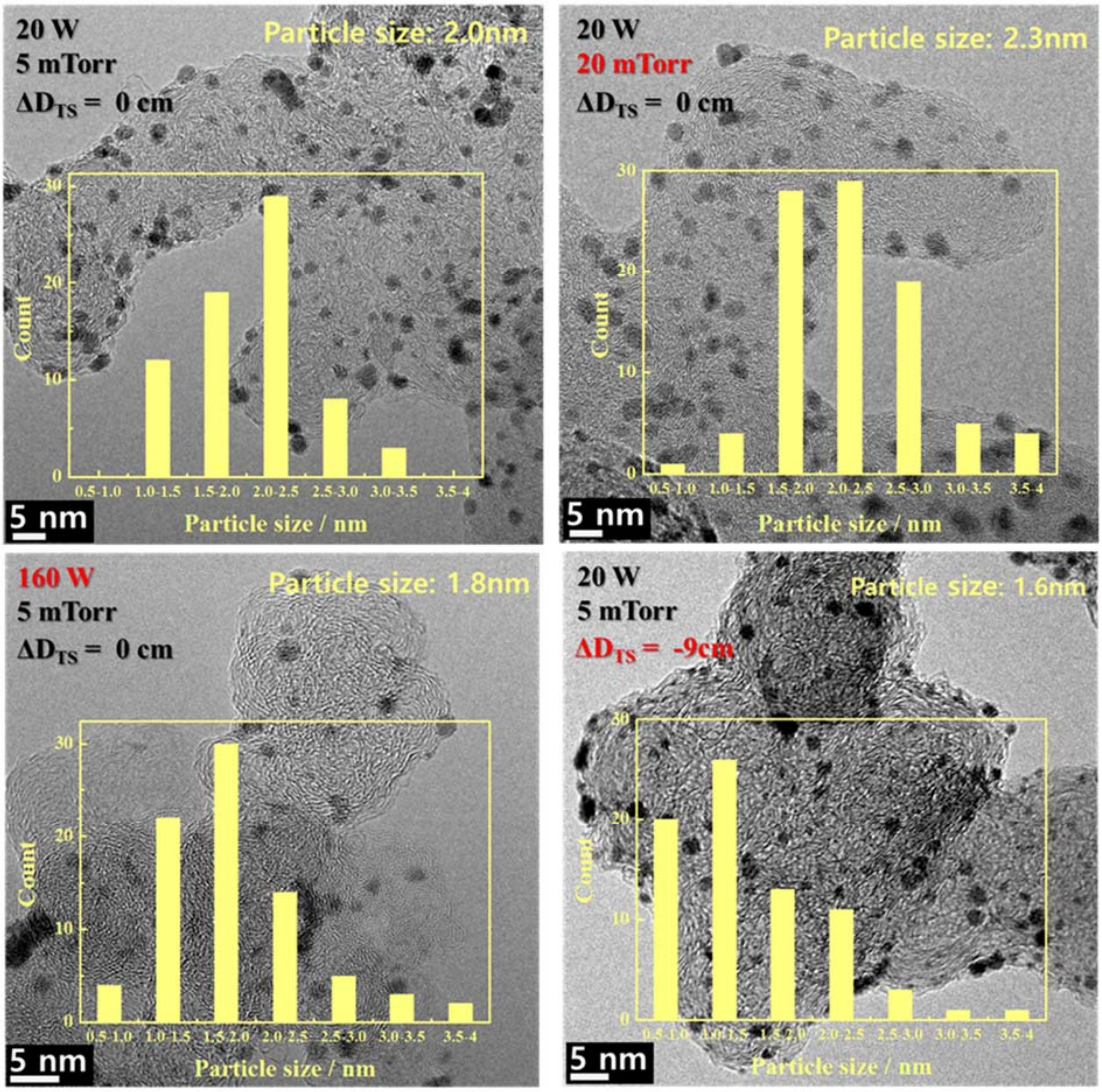

Supplementary Fig. 4. TEM images of Pt/C prepared by various sputtering conditions: (a) standard condition, (b) increased working pressure (20 mTorr), (c) increased sputtering power (160 W), and (d) decreased distance between target and Glu substrate (-9 cm).

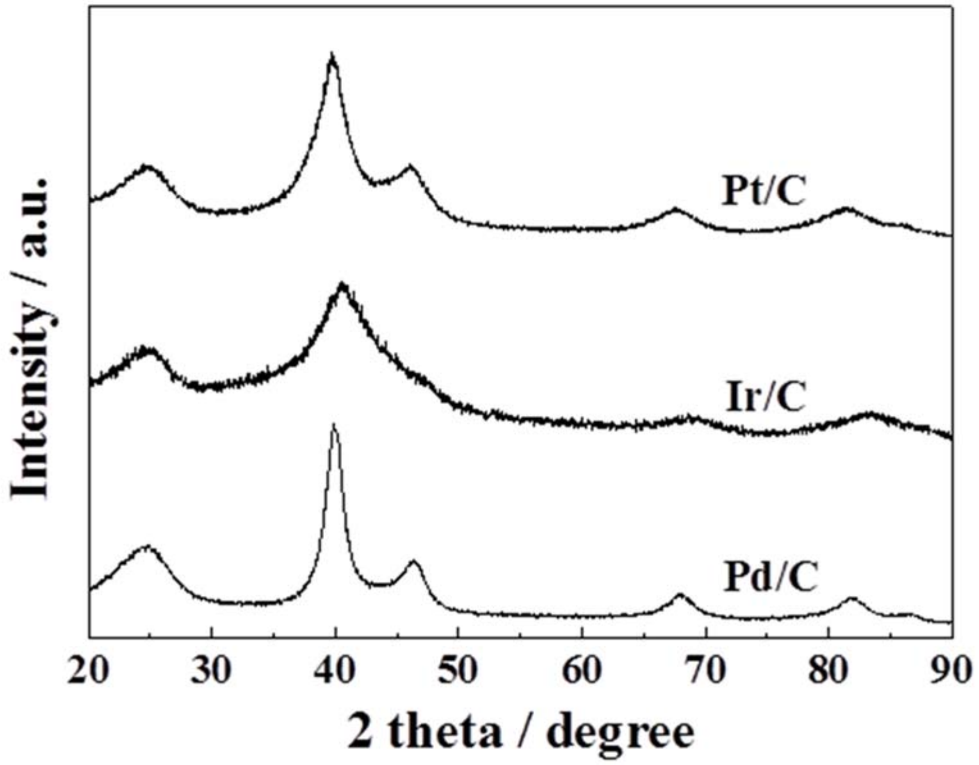

Supplementary Fig. 5. XRD pattern of Pt/C, Ir/C, and Pd/C.

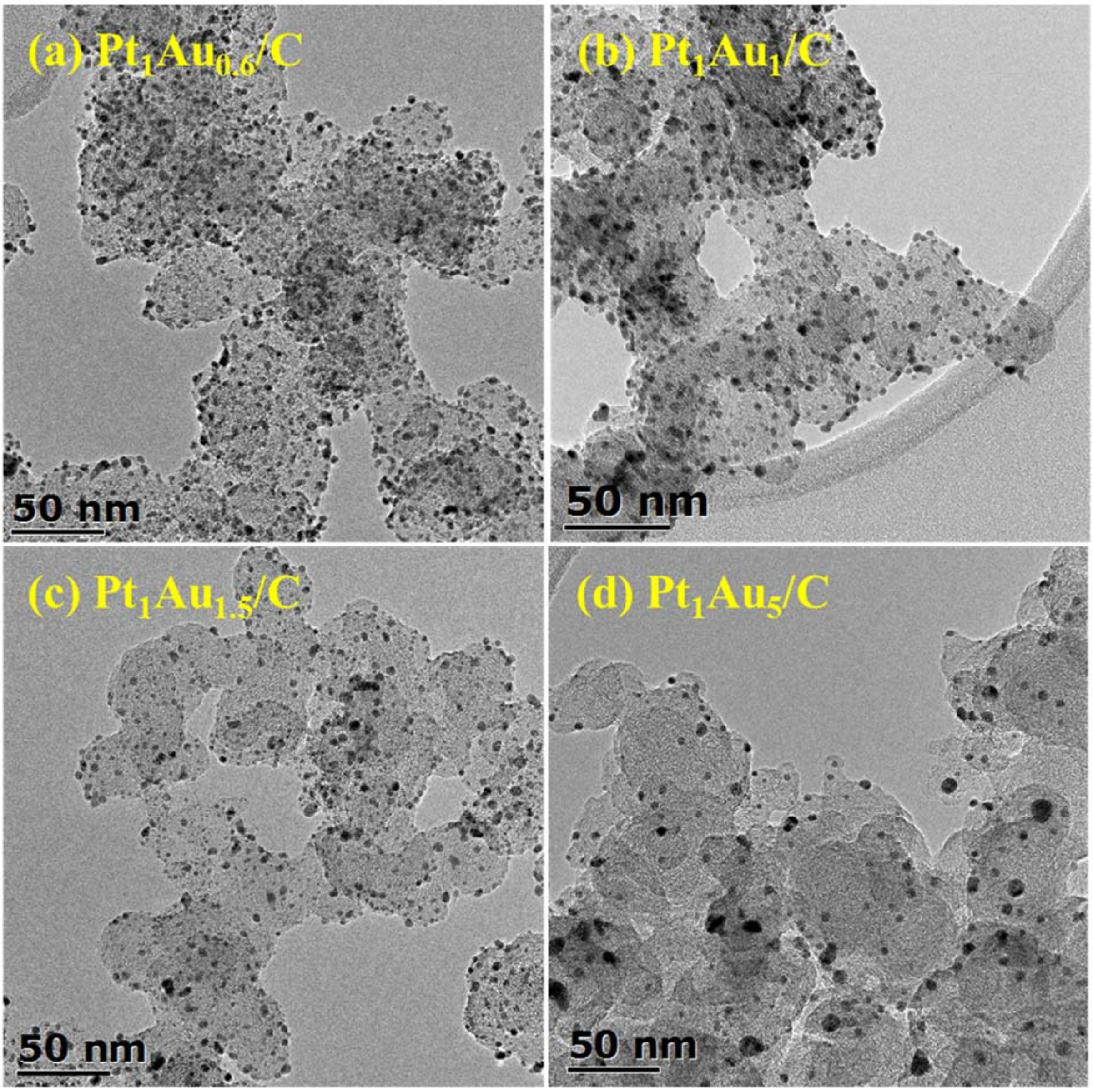

Supplementary Fig. 6. TEM images of Pt_xAu_{1-x}/C with various composition.

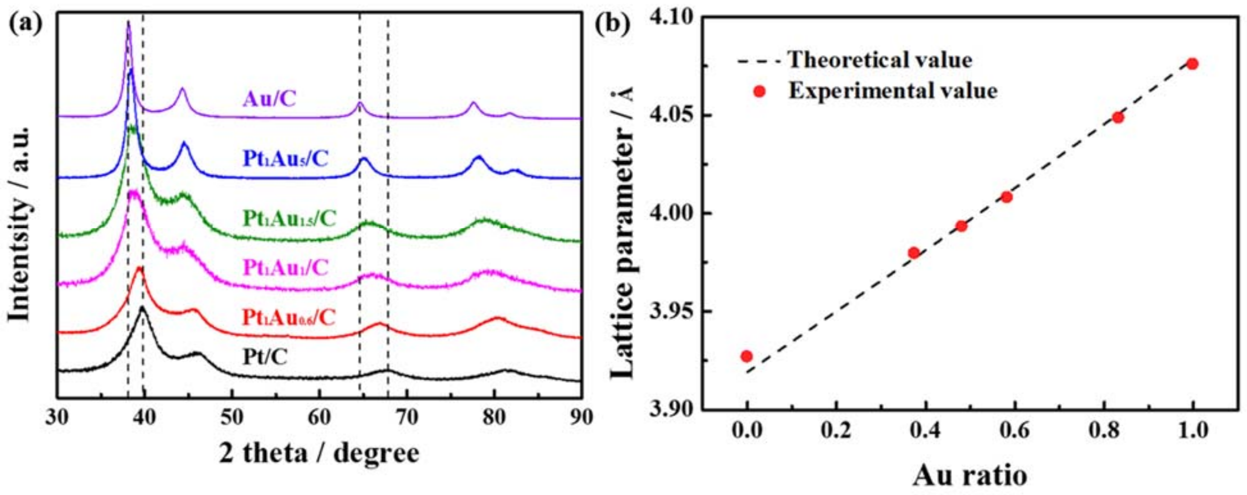

Supplementary Fig. 7. (a) XRD pattern of Pt_xAu_{1-x}/C and (b) Lattice parameter derived from XRD patterns (red dot) and calculated using Vegard's low (dashed line). The lattice parameters of Pt_xAu_{1-x} alloys NPs were calculated using Vegard's law and 2θ angle of (220) plane diffraction of Pt (67.45° from PDF# 65-2868) and Au (64.58° from PDF# 65-2870). The lattice parameter of the Pt_xAu_{1-x}/C were determined using the 2θ values of (220) plane diffraction in XRD pattern. The atomic composition of the Pt_xAu_{1-x}/C was analyzed using X-ray fluorescence (XRF) spectroscopy.

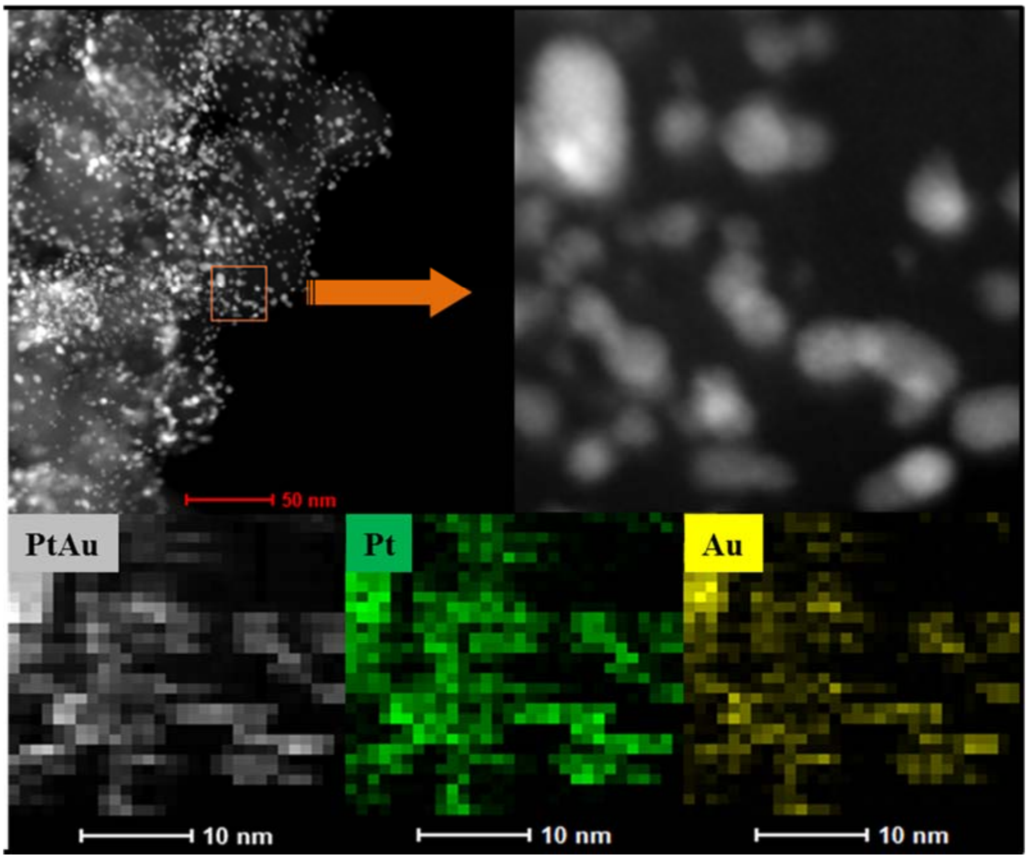

Supplementary Fig. 8. STEM image and color image obtained from EDX analysis of $Pt_{0.5}Au_{0.5}/C$.

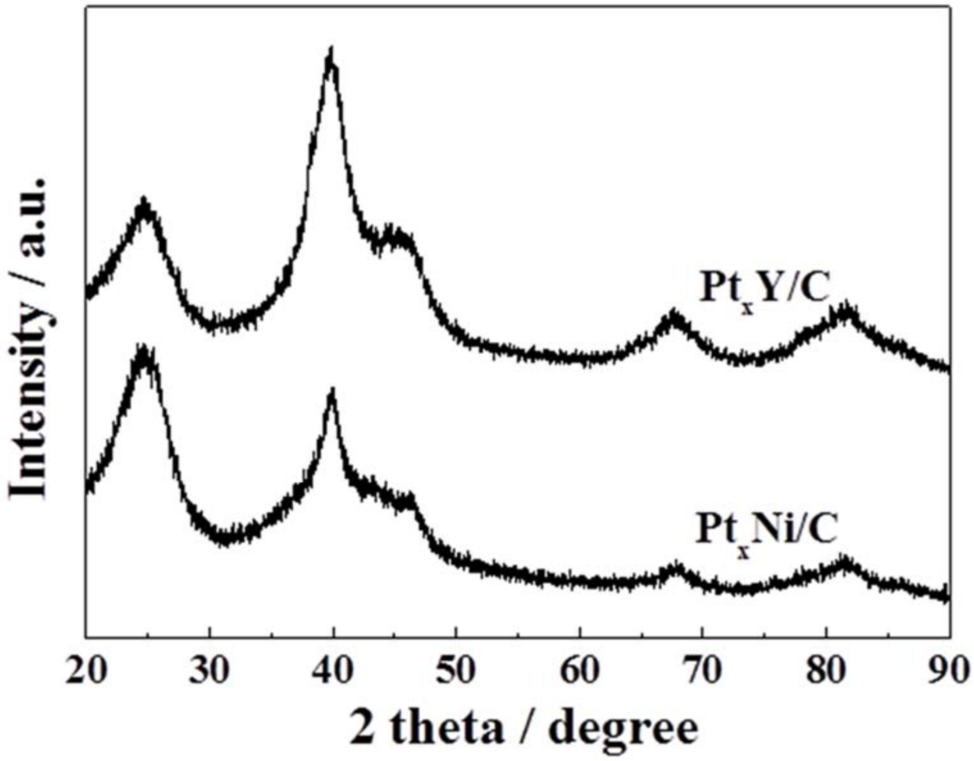

Supplementary Fig. 9. XRD pattern of Pt_xY/C and Pt_xNi/C.

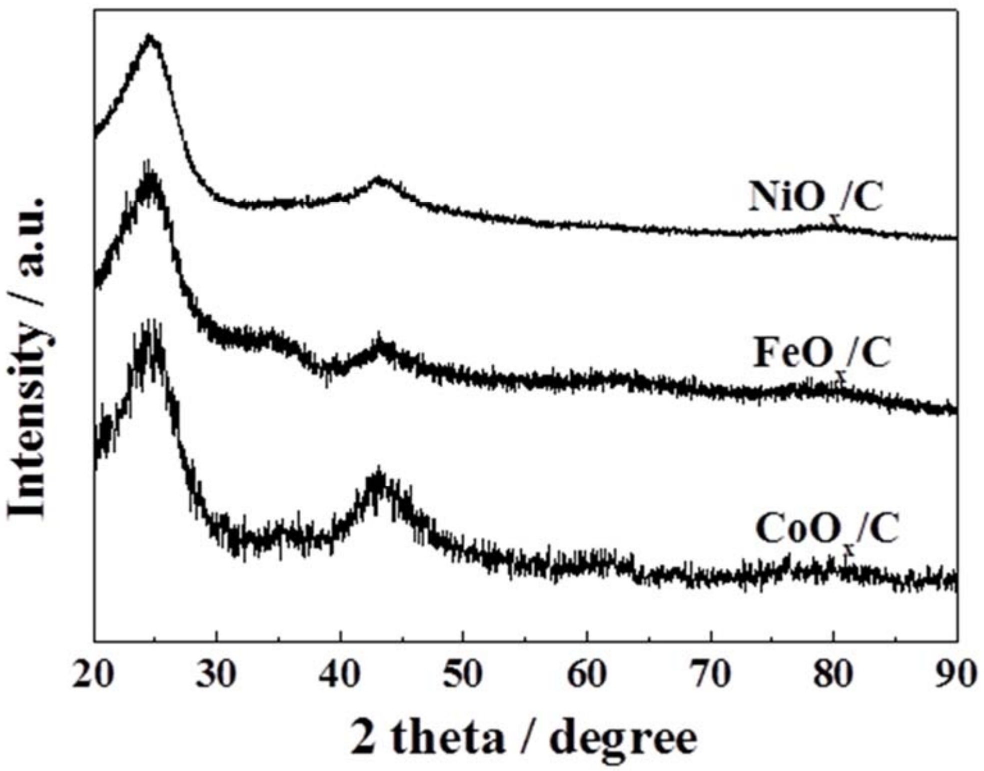

Supplementary Fig. 10. XRD pattern of NiO_x/C, FeO_x/C, and CoO_x/C. The diffraction of MOs were hardly detected in the XRD pattern likely due to finite size and/or low crystallinity of MO NPs.

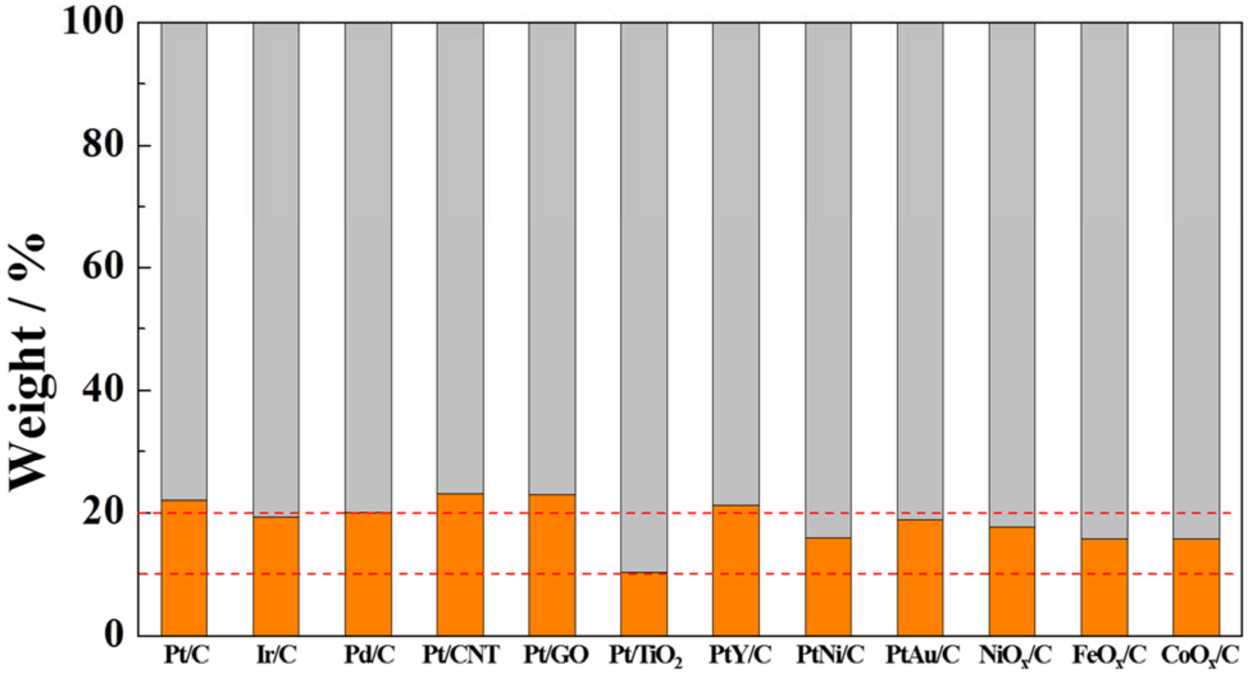

Supplementary Fig. 11. Weight fraction of NPs in the catalysts determined using the remained weight at the end of TGA (up to 900 °C, 5 °C min^{-1}) under an air atmosphere. The weight fraction of Pt in Pt/TiO_2 was determined using the ICP-MS analysis. All the samples were prepared to contain 20 wt.% of NPs, except Pt/TiO_2 (10 wt.%). The weight fraction of NPs in M/C, Pt/CNT, Pt/GO, and Pt/TiO_2 were in fair agreement with that expected (20wt.%). However, the remained weight of MO/Cs without calcination at 180 °C for 1 hour under an air atmosphere were less than 20 wt.% (around 16~ 17 wt.%) at the end of TGA likely due to the Glu on the MO surfaces.

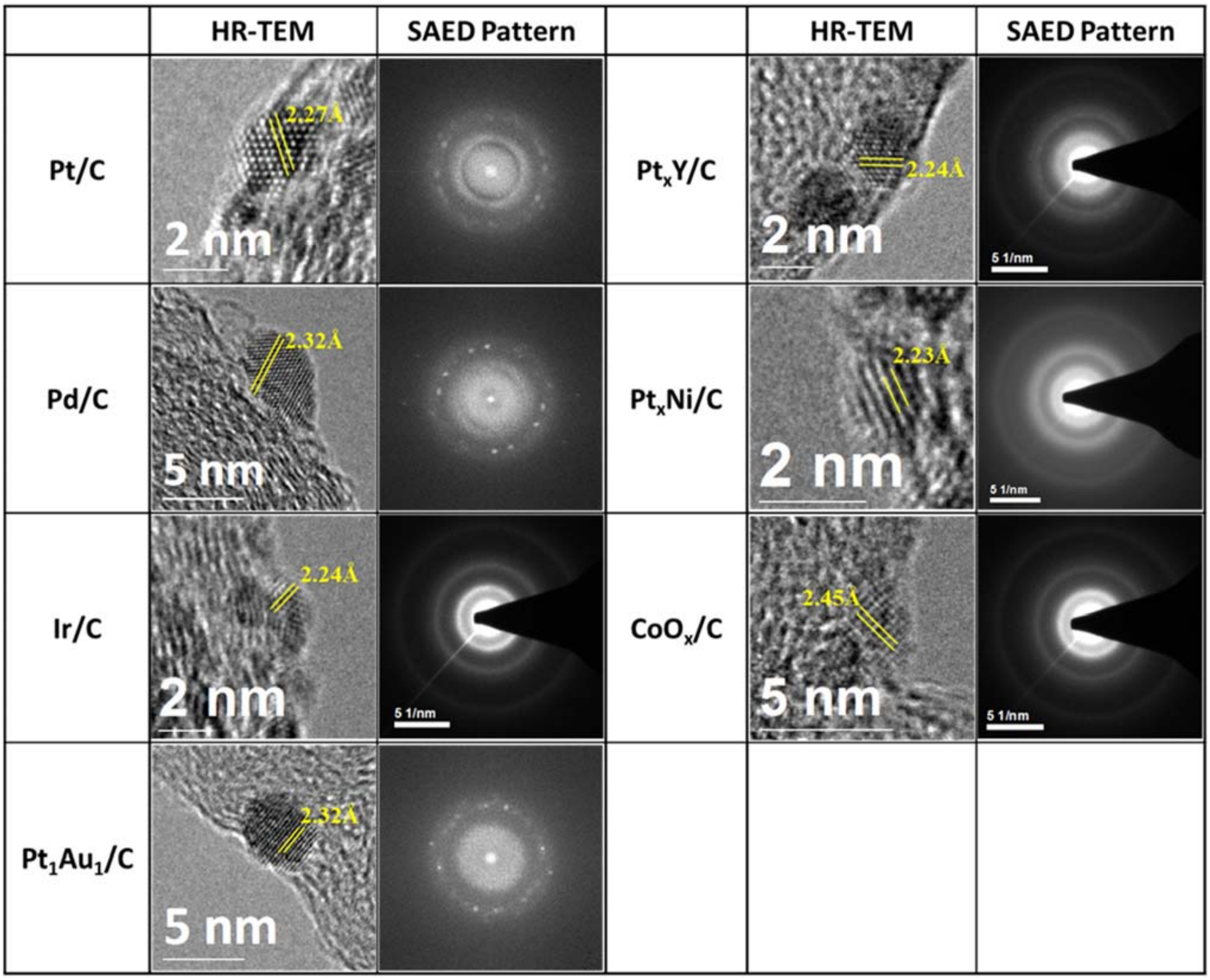

Supplementary Fig. 12. HR-TEM images of NP/C catalysts. The SAED pattern of Pt_xY/C, Pt_xNi/C, Ir/C, and CoO_x/C was given. The Furrier transform of HR-TEM images for Pt/C, Pd/C and Pt_1Au_1/C is also presented.

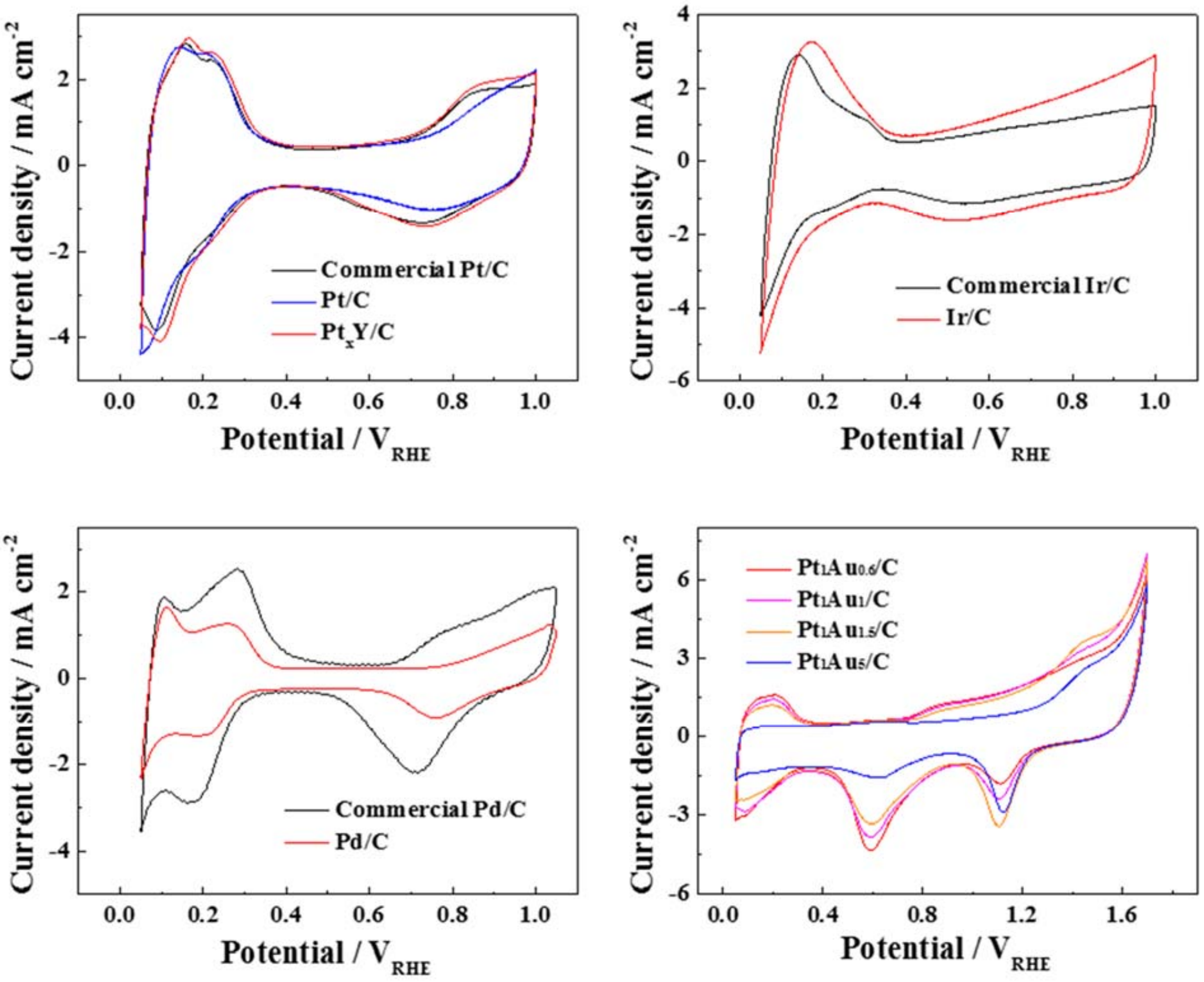

Supplementary Fig. 13. CV curve of M/C prepared using NP/Glu (red, blue, orange, pink lines) and commercial NP/C (black line).

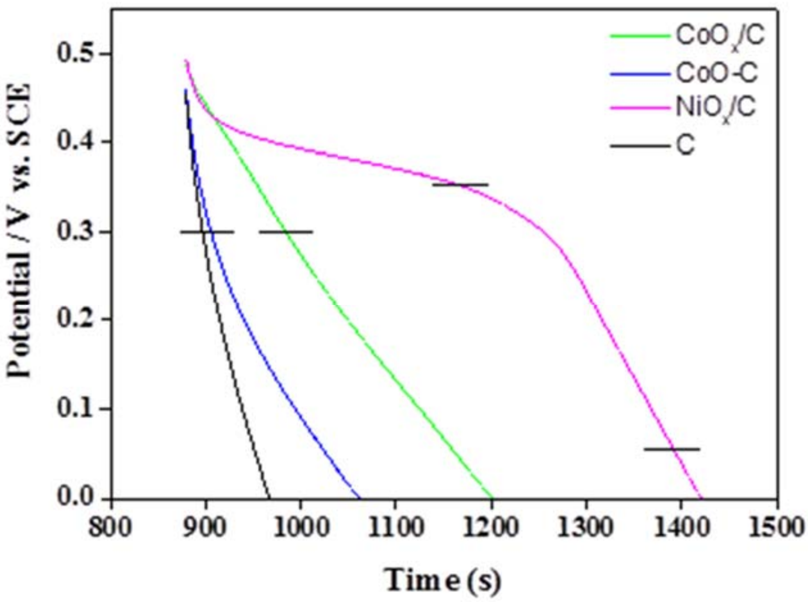

Supplementary Fig. 14. Discharge curve of CoOx/C, CoO-C, NiO$_x$/C, and C. The potentials range utilized to determined specific capacitance was indicated.

Supplementary Table 1. Electrochemically active surface area (EASA) of the catalysts.

Catalysts	EASA (m^2/g_{NP})	Catalysts	EASA (m^2/g_{NP})
Commercial Pt/C (20 wt.%, JM)	69.5	Commercial Ir/C (20 wt.%, Premetek)	53.3
Pt/C (21.9 wt.%)	64.7	Ir/C (19.3 wt.%)	68.0
Commercial Pd/C (20 wt.%, Premetek)	73.9	Pt_xY/C (21.3 wt.%)	69.1
Pd/C (20.1 wr.%)	41.5	CoO_x/C (15.8 wt.%)	231
$Pt_1Au_{0.6}/C$ (18.9 wt.%)	40.5	NiO_x/C (17.6 wt.%)	308
Pt_1Au_1/C (18.7 wt.%)	47.4	FeO_x/C (15.8 wt.%)	-
$Pt_1Au_{1.5}/C$ (19.2 wt.%)	40.8	Commercial CoO (Aldrich) on a thin film of carbon layer	3.4
Pt_1Au_5/C (18.9 wt.%)	2.6		

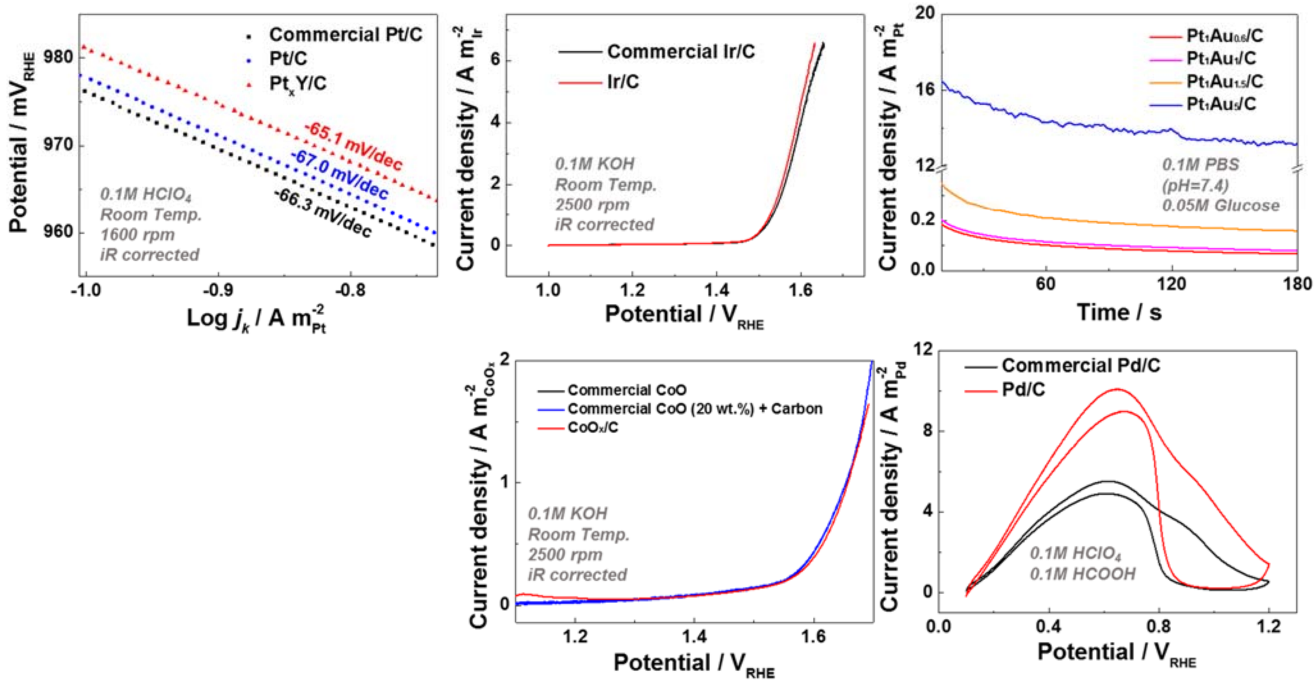

Supplementary Fig. 15. Current density normalized by EASA for electrochemical reactions.

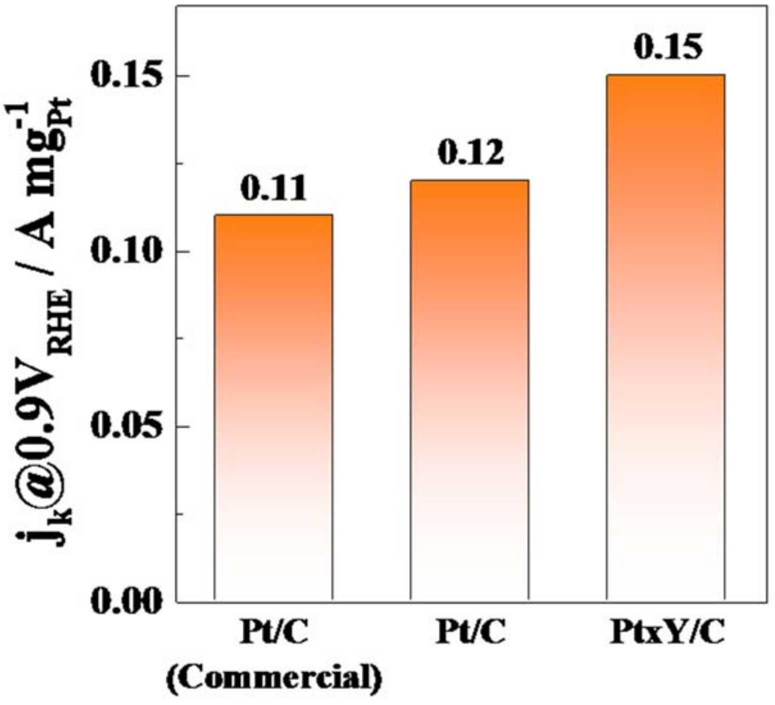

Supplementary Fig. 16. Mass activity for ORR on commercial Pt/C, Pt/C, and Pt_xY/C.

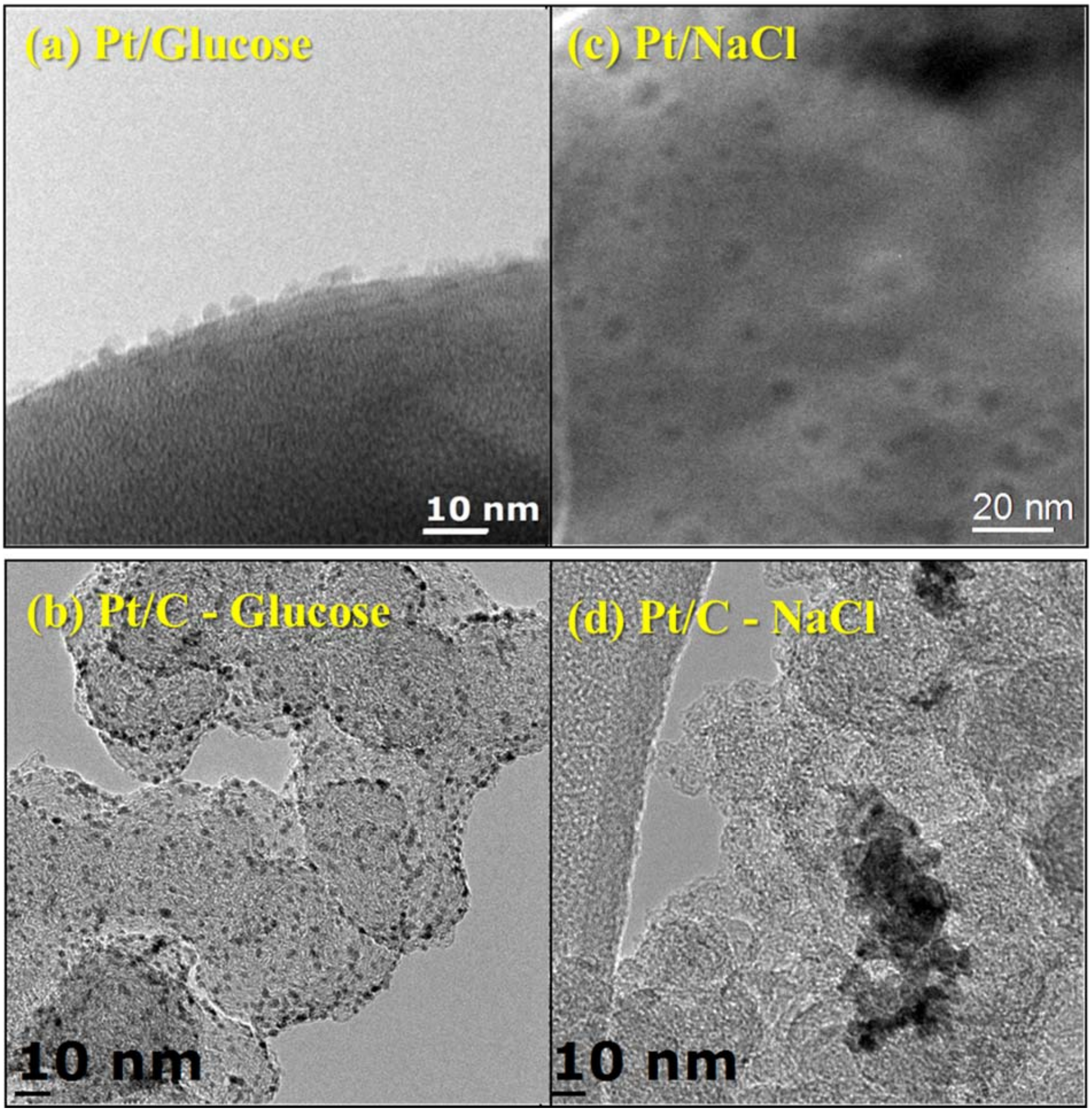

Supplementary Fig. 17. TEM images of (a) Pt/Glu, (b) Pt/C prepared using the Pt/Glu, (c) Pt/NaCl, and (d) Pt/C prepared using the Pt/NaCl.

Supplementary Table 2. XPS Curve fitting result (binding energy, area ratio) of commercial Pt/C (Pt/C (Pre)), Pt/CC prepared using the Pt/Glu (Pt/C (Glu)), Pt/Glu, and Pt/NaCl.

		Pt^0	$Pt(OH)_2$	Pt^{2+}	Pt^{4+}
Binding energy / eV	Pt/C (Pre)	71.7	72.7	73.6	74.9
	Pt/C (Glu)	71.7	72.7	73.5	74.7
	Pt/Glucose	71.5	72.5	73.6	74.8
	Pt/NaCl	71.6	72.6	73.5	74.7
Ratio / %	Pt/C (Pre)	46.3	22.8	13.8	17.1
	Pt/C (Glu)	46.0	21.3	15.6	17.0
	Pt/Glucose	5.2	49.2	21.6	24.0
	Pt/NaCl	57.4	22.4	10.4	9.8

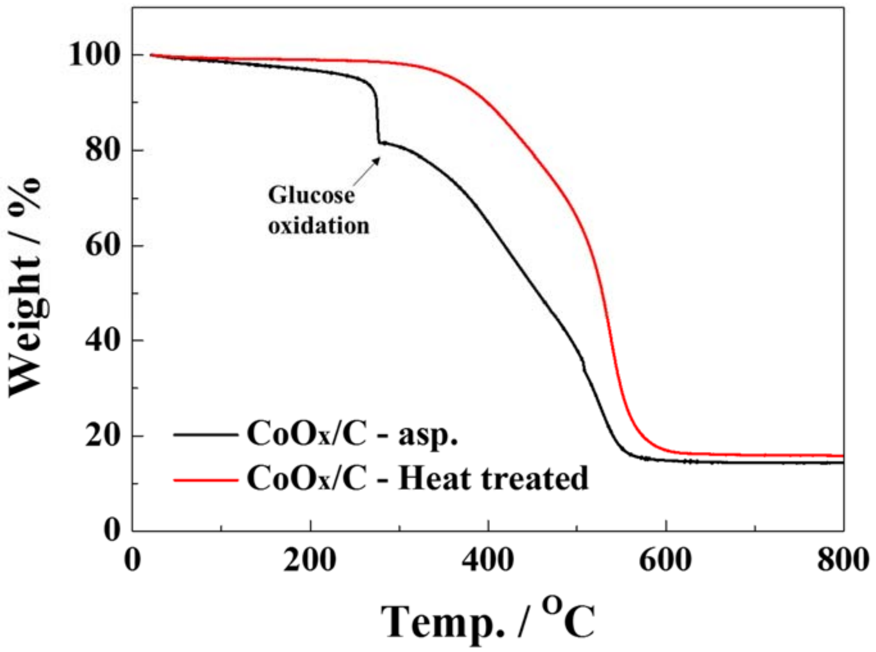

Supplementary Fig. 18. TGA curve of CoOx/C before and after calcination at 180 °C

Supplementary Table 3. Sputtering power for various catalysts.

Pt/C	Pd/C	Ir/C	Pt_xAu_Y/C, Pt: 20W				Pt_xY_Y/C		Pt_xNi_Y/C		M_xO/C (M= Ni, Co, Fe)
			Au								
			1:0.6	1:1	1:1.5	1:5	Pt	Y	Pt	Ni	
20W			11W	18W	24W	57W	40W	20W	20W	187W	180W

Materials

Commercial electrocatalysts (Pt/C, Ir/C, Pd/C) were purchased from Premetek Inc. and utilized without further heat treatment for the electrochemical analysis. 4-inch metal and metal oxide targets (Pt, Ir, Pd, Au, Ni, Y, NiO, CoO and FeO) were purchased from United Vacuum & Materials (Purity 99.99%). High surface carbon was purchased from Cabot co. (Vulcan XC-72R, BET: 237 m^2/g). TiO_2 nanopowder was purchased from Sigma Aldrich (particle size < 100 nm) and CNT was purchased from Carbon Nano-material Technology Co. The GO was supplied by Carbon convergence material research center in KIST Jeonbuk branch composite material technology institute and prepared using Hummers' method (J. Am. Chem. Soc., 80 (1958) 1339). The solvent of Nafion solution was a mixture of alphatic alcohol (85~80%) and water (15~20%).

Electrochemical analysis

Preparation of catalyst coated glassy carbon electrode. The catalyst layer was obtained as follows: (i) first, a slurry was prepared by ultrasonic agitation of a mixture of 800 μL of DI water, 10 mg of catalyst, and 60 μL of Nafion solution (Aldrich: 5 wt% Nafion) for 10 min; (ii) second, 5 μL of the slurry was pipetted and spread on the carbon disc; (iii) third, the electrode was dried at 60 °C for 10 min.

Oxygen reduction reaction (ORR) polarization

Prior to measuring ORR polarization curves, multiple cyclic voltammetry (CV) was carried out in deaerated 0.1 M $HClO_4$ solution at the can rate of 50 mV s^{-1} in a potential window of 0.05 - 1.0 V vs RHE, at room temperature. The ORR activity was evaluated by the rotating

disk electrode (RDE) technique in O_2-saturated 0.1 M $HClO_4$ solution with a sweep rate of 5 mV s^{-1} at 1600 rpm, at room temperature.

Oxygen evolution reaction (OER) polarization

OER polarization curve was recorded using cyclic voltammetry (CV) at a scan rate of 5 mV s^{-1} and 2500 rpm in N_2 saturated 0.1 M KOH solution. The OER activity polarization curve was stabilized after around 10th cycles of CV analysis and, thus, the OER activity was determined using the stabilized polarization curve. Following each measurement, 0 V vs. RHE was established by performing the hydrogen oxidation and hydrogen evolution reaction in the same electrolyte. An impedance spectrum was recorded with a peak-to-peak amplitude of 10 mV, at frequencies from 1 kHz to 500 kHz for evaluating the Ohmic resistance, which is obtained from the high-frequency intercept (or minimum) on the horizontal (real) axis of the Nyquist plot; typically, it was 31 Ω.

The OER activity of commercial CoO was measured using CoO particles deposited on a thin film of carbon support layers (CoO-C).To prepare a thin layer of carbon support (0.237 g cm_{geo}^{-2}), a suspension of Vulcan XC-72 (0.08 g) in 1,2-propanon (8.6 mL) was prepared. Then, 5 μL of the suspension was dropped onto the RDE electrode and dried in a gentle Ar stream. After drying, a 5 μL suspension of commercial CoO (0.02 g) in 1,2-propanol (8.6 mL) was pitted onto the carbon layer and dried. The amount of CoO was 0.059 g cm_{geo}^{-2}. Finally, a 5 μL diluted Nafion solution (10 vol.% in 1,2-propanol) was dropped and dried.

Glucose oxidation reaction

The activity of the Pt_xAu_{1-x}/C alloys toward electrochemical oxidation of glucose was determined using cyclic voltammetry (CV) and chronoamperometry (CA) technique. CV was

conducted in the potential range from −1.0 V to 1.0 V vs. RHE with a scan rate of 50 mV/s in an electrolyte composed of 0.5 M phosphate buffer solution (PBS, pH 7) and 0.05M glucose. CA was performed at 0.6V vs. RHE for 3600s. All of the electrolytes were purged with N_2 gas for 30 min to remove any dissolved gas prior to the electrochemical tests.

Formic acid oxidation reaction

The electrochemical activity of formic acid oxidation reaction (FOR) on Pd/C and commercial Pd/C were measured using cyclic voltammetry (CV) in a N_2-saturated 0.1 M $HClO_4$ + 0.1 M HCOOH solution at 50 mV s^{-1}.
